# How embedded is public involvement in mainstream health research in England a decade after policy implementation? A realist evaluation

**DOI:** 10.1177/1355819617750688

**Published:** 2018-04-13

**Authors:** Patricia Wilson, Elspeth Mathie, Fiona Poland, Julia Keenan, Amanda Howe, Diane Munday, Sally Kendall, Marion Cowe, Sophie Staniszewska, Claire Goodman

**Affiliations:** 1 Professor of Primary and Community Care, Centre for Health Services Studies, University of Kent, UK; 2 Research Fellow, Centre for Research in Primary and Community Care, University of Hertfordshire, UK; 3 Professor of Social Research Methodology, School of Health Sciences, University of East Anglia, UK; 4 Research Fellow, School of Health Sciences, University of East Anglia, UK; 5 Professor of Primary Care, Norwich Medical School, University of East Anglia, UK; 6 Lay Member, Public Involvement in Research Group, University of Hertfordshire, UK; 7 Professor of Community Nursing and Public Health, Centre for Health Services Studies, University of Kent, UK; 8 Professor of Patient and Public Involvement and Experiences of Care, RCN Research Institute, University of Warwick, UK; 9 Professor of Health Care Research, Centre for Research in Primary and Community Care, University of Hertfordshire, UK

**Keywords:** health research, patient and public involvement and engagement, realist evaluation

## Abstract

**Objectives:**

To explore how embedded patient and public involvement is within mainstream health research following two decades of policy-driven work to underpin health research with patient and public involvement in England.

**Methods:**

Realist evaluation using Normalization Process Theory as a programme theory to understand what enabled patient and public involvement to be embedded as normal practice. Data were collected through a national scoping and survey, and qualitative methods to track patient and public involvement processes and impact over time within 22 nationally funded research projects.

**Results:**

In research studies that were able to create reciprocal working relationships and to embed patient and public involvement this was contingent on: the purpose of patient and public involvement being clear; public contributors reflecting research end-beneficiaries; researchers understanding the value of patient and public involvement; patient and public involvement opportunities being provided throughout the research and ongoing evaluation of patient and public involvement. Key contested areas included: whether to measure patient and public involvement impact; seeking public contributors to maintain a balance between being research-aware and an outsider standpoint seen as ‘authentically’ lay; scaling-up patient and public involvement embedded within a research infrastructure rather than risk token presence and whether patient and public involvement can have a place within basic science.

**Conclusions:**

While patient and public involvement can be well-integrated within all types of research, policy makers should take account of tensions that must be navigated in balancing moral and methodological imperatives.

## Introduction

While patient and public involvement (PPI) in research is increasing in many parts of the world,^1^ the United Kingdom (UK) health research arena is recognised as having led the way through its significant policy drive to embed PPI within the national health research infrastructure.^2^ PPI became a statutory part of the national research governance framework in 2005 and is now integral to the main UK health research funding streams. Researchers are routinely required to show how PPI has shaped their research proposal and its delivery.

To establish PPI within a national research infrastructure requires significant investment. Such resources are commonly justified by two main arguments. First, alongside the democratic imperative, the moral argument asserts that research conducted on people without their input is unethical. The second argument for PPI is methodological, that having PPI within a research study will improve recruitment, impact and outcomes.

Despite claims for impact, there is less evidence of the actual processes or mechanisms that enable PPI to fulfil its promises. Ten years after a PPI research infrastructure was created in the UK, it is also unclear whether and how PPI has become embedded within the health research environment. This paper presents findings from a realist evaluation of PPI in health research in England^3^ to explore how PPI becomes integrated within clinical research and what actions enable this.

The terms ‘participation’, ‘engagement’ and ‘involvement’ are often used interchangeably to capture what PPI entails. For the purposes of this paper, we use the following definitions: ‘involvement’ being active involvement of public members in advising on scope, direction and conduct of research and research organizations; ‘engagement’ involving the provision and dissemination of information and knowledge about research and ‘participation’ denoting when people take part in a research study to provide its data.^2^

## Methods

The Research into Patient and Public Involvement: A Realist Evaluation (RAPPORT) study^3^ was conducted in England from 2011 to 2013 and was approved by the National Research Ethics Service Committee East Midlands – Nottingham 1 Research Ethics Committee (reference 11/EM/0332).

Previous evaluations of PPI have been criticised for using designs incapable of exploring contextual factors and mechanisms that enable or inhibit PPI process and impact.^4^ We adopted a realist evaluative design^5^ to develop a theory-driven account of what enables PPI to be integrated as normal practice, and under what circumstances, in terms of research type and setting.

**Figure 1.**
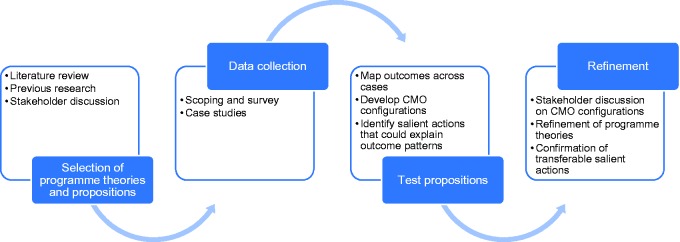
RAPPORT study design (adapted from Pawson and Tilley^5^ and Hewitt et al.^7^). RAPPORT: Research into Patient and Public Involvement: A Realist Evaluation. CMO: context, mechanism and outcome.

The RAPPORT study design is illustrated in Figure 1. Data were collected through two main methods. First, a national scoping exercise and survey was conducted with investigators of studies adopted by the UK Clinical Research Network, detailed elsewhere.^6^ The results were used to select 22 nationally funded research projects as case studies in which to explore PPI processes and impact. Case studies were followed up over 18 months, through semi-structured interviews (initial and regular follow-up) and documentary analysis (for example, notes from team meetings).

As we were interested in how PPI becomes embedded within clinical research, Normalization Process Theory^8^ provided an explanatory theory to inform the development of a PPI specific programme theory about how PPI has become embedded (or not) as normal practice within health research.^9^

To capture a broad range of study designs, populations and topic areas, we purposively focused our data collection on six diverse topic areas: arthritis; cystic fibrosis; dementia; diabetes; intellectual and developmental disabilities and public health (Table 1). This ensured that we included studies with different population ages, accessibility challenges and history of PPI. Cystic fibrosis studies were more likely to be laboratory-based or have a biomedical focus, whereas the other topic areas tended to involve a range of designs and sample size.

**Table 1.** RAPPORT case studies.

Topic	Study design	Funder	Documents	Interview participants
Dementia	Basic science involving humans	National Institute of Health Research (NIHR)	38	3
Dementia	Clinical Trial of an Investigational Medicinal Product (CTIMP)	NIHR	5	4
Diabetes	CTIMP	NIHR	19	4
Diabetes	Trial, cohort, qualitative	NIHR	13	9
Diabetes	Mixed qualitative/quantitative	NIHR	8	4
Diabetes	Qualitative	Non-commercial	1	3
Diabetes	Randomized Controlled Trial (RCT) to compare intervention	NIHR	11	4
Diabetes	Basic science involving humans	Charity	16	4
Diabetes	Genetic epidemiology	Charity	9	3
Diabetes	Intervention (mixed quantitative/qualitative)	Charity	4	10
Arthritis	CTIMP	Charity	3	4
Arthritis	Research database	Research Council/Charity	0	4
Arthritis	Cohort	Charity	3	9
Arthritis	Qualitative methods	Research Council	16	4
Public health	RCT to compare intervention	NIHR	5	4
Public health	RCT to compare intervention	NIHR	27	6
Public health	Survey/qualitative	Charity	19	13
Public health	RCT to compare intervention	NIHR	12	6
Intellectual and developmental disabilities	Questionnaire	NIHR	17	4
Intellectual and developmental disabilities	RCT to compare intervention	NIHR	9	7
Intellectual and developmental disabilities	Systematic review	NIHR	22	6
Cystic fibrosis	CTIMP	NIHR/Research Council	21	4
Total			278	119

RAPPORT: Research into Patient and Public Involvement: A Realist Evaluation.

Within the case studies, we interviewed 64 researchers, 48 public contributors, 7 PPI coordinators and 10 representatives from funding organizations and UK Clinical Research topic networks (n = 129). We also analysed 278 documents. We were more successful in recruiting interviewees from some topic areas than others, and case studies differed in providing us access to their documents.

**Figure 2.**
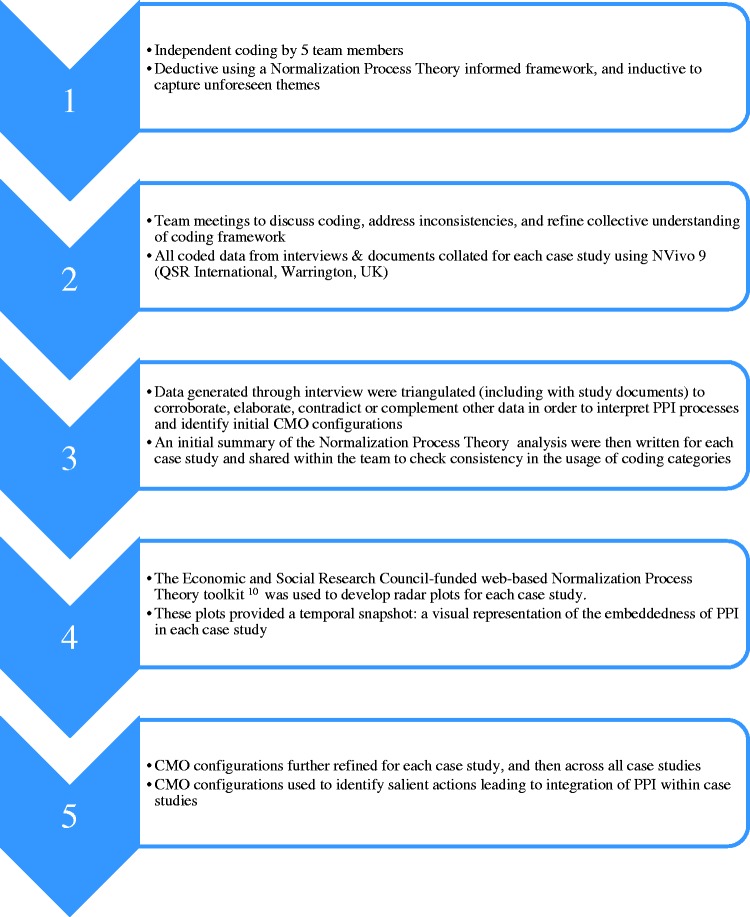
Approach to analysis.

We used Normalization Process Theory to provide an initial coding frame for the analysis of the interview data and documents.^10^ We followed a stepped approach to data analysis^11^ (Figure 2) and the study itself was underpinned by PPI as reported elsewhere.^12^

The use of Normalization Process Theory radar plots^10^ and realist evaluation summaries helped us understand changes in PPI processes over time within case studies. Regular research team meetings were held to compare data across the case studies and discuss emerging context, mechanism and outcome (CMO) configurations. These CMO configurations were tested across the data set and rejected, accepted, or refined. Candidate CMO configurations were further refined through discussion with case study participants, stakeholder reference groups made up of public contributors, and the RAPPORT independent advisory group. Finally, the configurations were presented at four regional events in England for refinement.

## Results

We identified six CMO configurations and salient actions^13^ that could explain what enabled PPI integration within the case studies.

### Salient action 1: a clear purpose, role and structure for PPI are ensured

A clear structure and understanding of the different roles was required if PPI was to be seen as a different way of working with patients and the public. Requirements included an identified person to coordinate PPI, but also the whole team being supportive of PPI, with the work required for PPI being appropriately allocated. Providing skills, continued support and trust in each other’s input were also key mechanisms.

This salient action was heavily influenced by the non-negotiable requirement of UK research funders that grant applications detail how PPI had contributed to the proposal and would be operationalised within the project. Many researchers had engaged with PPI because they ‘had to’ to obtain funding. There were differences between the main funding bodies on how they prioritised and understood PPI. The funding programmes and management organizations of the English National Institute for Health Research (NIHR) suggested that PPI impact should mainly be seen from the methodological perspective, with PPI as a way of improving research quality.

It [PPI], you know, is likely to improve recruitment. It makes sure that it meets patient needs so that when it gets out the other end into the implementation phase it can actually have a possibility of having impact. (Funder, NIHR)

In contrast, the large charitable funders often prioritised the moral imperative for PPI.

…we get our money from the public, from people with the condition, so it’s really important that we include viewpoints of people with the condition in our decision. (Funder, Charity)

This initial funder-led coercion could be gradually transformed into research team enthusiasm with the positive impact of PPI. Funders spoke of a ‘sea-change’ and some attributed a perceived improvement of the quality of funding applications to growing PPI contributions over the past 10 years.

The growth of PPI-related activity within funding organizations had also brought about what one respondent described as ‘industrial scale PPI’, seeming perhaps at odds with the person-centred ethos of the moral perspective of PPI. One consequence of this formal PPI activity was the need to have people responsible for coordination and continuity of PPI within research. This had created a new role with a focused responsibility for coordinating PPI. This role was often challenging and demanded much effort, particularly if dealing with large numbers of public contributors through email or social media.

For research teams without dedicated PPI support, access to an external pre-existing PPI group was an enabler. With the drive towards PPI within the NIHR infrastructure, topic-specific PPI panels had developed. Some researchers stated a preference for this outsourced approach, suggesting it provided access to a wider, more representative group, than the ‘usual suspects’ who were part of more local groups. In contrast to working with an ‘in house’ group, the interaction between the outsourced PPI panel and research team tended to be for a single task; for example, reviewing patient recruitment literature. While the purpose and role for PPI was very clear in these circumstances, it also limited PPI impact to discrete stages of the research process. In contrast, studies with embedded PPI extended impact to, for example, dissemination and identification of further research priorities.

### Salient action 2: active recruitment of public contributors who reflect the diversity of a study population

Researchers and funders acknowledged that to ensure public involvement that reflected the defined research study population posed a significant challenge. While training in community engagement was helpful, study teams located within the study population (for example, clinical study teams) found it easier to recruit public contributors. However, we found unintended consequences of this approach to PPI recruitment, with evidence of some merging of roles between being study participant and also of being asked to advise the study team. This dual role provided helpful consumer feedback (for example, how the process of data collection was experienced) and could work well, but was potentially problematic if participant and advisor roles were blurred. Patients undertaking this dual role were further confused when the investigator was also their clinician; reporting it was difficult to remember whether they were talking to the investigator as a participant, patient or advisor.

You have to switch your hat and say ‘Look I’m not a patient expert today, I’m a patient of yours and I want you just to look at my illness and not discuss any other research’. (Public contributor)

We also found examples of a single public contributor acting as a link to the broader community. Frequently, these people were recruited from charities that served the study population. This worked well when the single representative had strong links with the relevant community and acted as a two-way conduit between the research team and community. However, recompense for their time was sometimes an issue, particularly when the PPI work impinged on other roles they had within the charity.

### Salient action 3: whole team engagement with PPI

For this action to be achieved, and despite new roles being created, all researchers in the team needed nonetheless to engage with PPI. While having someone in the research team responsible for PPI was important, not having senior researchers engaged could lead to tokenistic PPI. We found that the need to explain technical aspects of the research to public contributors had also increased engagement with PPI throughout the research team.

In 2014, producing a plain English summary of a research project became a mandatory part of the application for NIHR funding. Researchers reported being initially challenged by this, but also described how working with public contributors had helped, again supporting a visible positive impact of PPI. Having the ability to write a publicly accessible explanation of a research study was identified by senior researchers as a transferable skill useful across their range of work. When there was whole team engagement, recognizing the full impact of PPI within projects was also more likely through a shared narrative.

### Salient action 4: mutual understanding and trust

Strong relationships were found to be fundamental to PPI and these are predicated on a shared understanding of what PPI is. There was some evidence that researchers, and in particular, newer researchers, did not fully understand the difference between patients participating in research as a ‘subject’, and being involved as advisors. The more junior researchers had often joined a study only once it was funded and received minimal training on PPI as part of their general research education. In contrast, more senior researchers understood the concept of PPI because of the discipline of meeting funding requirements and having been involved in applying for funding.

Trust also underpinned relationships in PPI and was seen by researchers as essential for the smooth running of the study. For public contributors, trust was created when they felt valued. This sense of value was enhanced if there was explicit appreciation by the research team of their input, but especially if the relationship was ongoing, ensuring that even at the end of the study there was feedback given on how their input had influenced the study. To this end, the PPI arena (its physical setting, social context, and formality) where their input was aired, needed careful facilitation. In more formal settings, such as trial steering committees, public contributors needed a skilled chairperson who ensured that their contributions were made within an equitable environment where all perspectives and needs were recognized and valued.

Trust was also engendered by a sense that there was fair reimbursement of PPI time. All case studies reimbursed for ‘out of pocket’ expenses; however, reimbursing for time varied. Complexities around payment to people while they were in receipt of social welfare was raised as one significant issue, but some public contributors expressed unhappiness with the level of payment:

…people just make an assumption, oh they’re patients, we won’t need to pay them. And you know our time in our life is just as … important as a researcher’s.… (Public contributor)

While shared understanding and feeling valued were the foundations of relationships in PPI, the process of building and sustaining relationships needed nurturing over time. Only 19% (n = 9) of public contributors we interviewed were working with researchers they had not met or worked with before. Public contributors who were known to the team were actively recruited to new studies, bypassing the initial time required to establish the working relationship. Sustained engagement was also enabled by PPI groups being attached to research centres.

### Salient action 5: opportunities for PPI throughout the research process

It was common for public contributors to be involved throughout the research process within applied health research. However, some researchers did question whether this broad involvement was possible in basic science studies and had the potential to ‘jeopardize the research’ through a lack of technical understanding. There were examples where PPI had worked well in basic science research, including involvement in funding panels, and prioritizing studies for access to a tissue bank database. However, this type of involvement in basic science research required a dedicated facilitator and the resources to support this. For studies of any type without this level of support, public contributor involvement was most marked during project set-up but tailed off during recruitment phases when research teams had intense periods of work and hence less capacity to work with public contributors. Some public contributors described ‘losing contact’ with the project after extensive involvement during proposal development and study set-up.

To ensure involvement at all stages of the research cycle and as a way of addressing power differentials, it is increasingly common for a member of the public to be a co-applicant on a research grant application. For example, the NIHR application process allows for a ‘lay’ co-applicant’s CV to be submitted alongside those of the professional members of the research team. However, there were some concerns from laboratory-based scientists that having a lay co-applicant was now seen as the ‘gold standard’, and grants without one were seen to be less likely to succeed. These researchers felt that it would be very difficult to have a ‘non-scientist’ who would be able to be fully engaged in the project.

### Salient action 6: reflection, appraisal and evaluation of PPI

We found that ongoing evaluation was necessary for PPI integration as processes were modified to address any issues. However, only two of the case studies were systematically appraising PPI in their studies.

There were two reasons why studies did not evaluate their PPI. Some questioned why focus only on PPI and not the research process as a whole, and others identified the lack of readily available heuristic tools to support evaluation. However, many case studies did routinely record PPI activities.

The need for robust PPI evaluation to know whether it had an impact or not was seen as the ‘holy grail’ by some researchers. There was also some debate on what outcomes were being evaluated: PPI impact on the research study outcome (for example, recruitment of participants), or outcomes for researchers and PPI contributors themselves? A number of researchers commented on the impact of PPI in making them reappraise the way they viewed an issue, and how they related to the public. Public contributors frequently reported improved self-worth, increased health knowledge and respite from caring roles, as the personal impact of undertaking a PPI role.

### Barriers to normalizing PPI

While there was a direct relationship between the six salient actions and how integrated PPI was within a study, we also found a number of barriers to normalizing PPI. In global comparisons, PPI in UK health research can be seen as the most heavily shaped by the mainstream funders’ requiring PPI. Within RAPPORT and other studies,^14^ the influence of funders was seen to be clearly shaped by values which reflected a dichotomy between primarily methodological and primarily moral imperatives for PPI. This presents us with two discrete lenses through which to view barriers to PPI integration and the ongoing debate influencing how PPI may be embedded in health research.

First, there are disagreements between those who see developing tools to measure the impact of PPI as vital within a resource-constrained system, and those who see attempts to measure PPI impact as being at odds with understanding PPI as a moral right and process.^15^ While the scientific community expects robust evidence of the effectiveness of PPI, its complexity as a phenomenon makes it difficult to develop validated tools that can provide broadly meaningful evidence.^4,16^ One challenge is to accommodate the multiple perspectives involved, while acknowledging that these will influence what may be identified as important to evaluate.^17^ Some call for the impact of PPI not just to be seen as confined to the research outcomes but also to ways in which it has changed the perspectives of researchers^4^ and public contributors.^18^ Rose^19^ critiques current attempts at evaluating PPI for limiting evidence presented to descriptive case studies, which largely exclude information on impact more relevant for assessing how PPI changed the knowledge produced and its consequent impact on the ultimate research beneficiaries. Nevertheless, we found studies, which had embedded PPI, were matching their evaluation approach with the aims and purpose of PPI within that particular study, as endorsed by Edelman and Barron.^15^ Understanding how values shape the PPI purpose within a study, and so affect how PPI should be measured or evaluated, appears to be a prerequisite of transparency in any adequate evaluative approach.^20^

A second contested area in PPI is how to find ‘the right people’ to be public contributors. Public contributors have mainly been drawn from the well-educated, retired and affluent,^21^ and are so less likely to represent under-served populations. Some social anthropological perspectives would define the challenge as to find public contributors whose views authentically reflect their insider knowledge of the study population.^22^ However, this insider status can pose problems, not least because insiders from any community are still unlikely to be a homogenous group, and they will have their own diverse agendas not necessarily reflecting the range of views of the broader study population.^22^ Whether any public contributor can speak for an entire study population must therefore be debatable.^23^ However, others argue from a consumerist perspective that even without complete representativeness, at least some alternative perspectives will be voiced.^24^ Thus, recognizing the problem of representativeness informs a current emphasis on recruiting more diverse public contributors.^15^ The RAPPORT findings found that pre-existing relationships, which take time to develop, will encourage trust between researchers and public contributors. Timescales are often short for carrying out funded research, so it is unsurprising that most research teams within RAPPORT recruited patient and public contributors already known to them. However, there was evidence that this called for some teams to carry out a delicate balancing act in ensuring that the public involved had enough (research) knowledge to be able to contribute in a way researchers felt was useful, but not so much (research) knowledge that they lost their distinct perspective and became research team insiders. Maintaining this fine line is reported elsewhere^25,26^ and represents a fundamental conundrum of PPI: maintaining a perspective as an insider (to study population) and also as an outsider (to research team) over a study’s lifetime.

Within the case studies, we found some evidence that PPI was being ‘procured’ from external provider oganizations with whom the research team had little other engagement. The UK policy imperative for PPI in research has led to a rapid scaling-up, perhaps indicating a move towards an ‘involvement industry’^23^ characterised by commodification and standardization. A commodity can have both use and exchange values in transactions and PPI can be described as being ‘purchased’ through some exchanges of values which can improve the quality of research directly experienced and in its wider applications.^15,26^ Commodification in health care has been extensively critiqued as characterizing depersonalization and bureaucratic control.^27^ However, Timmermans and Almeling^27^ argue that commodification in the health care arena, rather than informal ad hoc arrangements, can be more effective in advancing important goals. Thus, it is not commodification as such that is the problem, but rather, the way it happens. Commodification may, equally, be interrelated with altruism and so engender new ideas and awareness. In those case studies exemplifying embedded PPI, a transaction was maintained between researchers and public contributors through experiential knowledge being treated as having value, and so facilitating reciprocity in terms of increasing public contributors’ self-worth alongside any monetary exchange.

Some suggest that requiring PPI at every research stage irrespective of whether the research focus is basic science or applied, or its stage of development, is a tyranny.^26^ We found in RAPPORT that basic science studies were finding unexpected benefits from PPI, and our interviews with funders revealed that after hesitant implementation, public involvement in ‘blue skies’ research funding committees had been less challenging than anticipated, bringing benefits such as more critical appraisal of the likely usefulness of the end product of translational research. This contradicts findings in the van Bekkum and Hilton^14^ study of UK research funding bodies’ views on PPI where some funders view PPI as unnecessary in ‘high-level science’. Those less critical of PPI in all health research still caution that PPI may at least be more challenging in basic science,^28^ and that levels of PPI needed will vary between topics and situations.^29^ Nevertheless, Callard et al.^30^ call for PPI to be reconceptualised within all stages of translational research. They warn that restricting involvement to latter stages, concerned only with improving recruitment to trials and to disseminating findings risks the research community producing interventions and products not fit for purpose, so that ethical concerns around new research areas, such as biomarkers, will not be interrogated, and omit patient and public potentially valuable contributions to early stages of the research process. As in considering commodification, we argue that the problem is not about involving public contributors at all stages and types of research, but that it is the less robust processes of involvement that more often cause issues.

## Discussion

Alongside a growing body of evidence is 20 years’ experience of attempts to embed PPI in UK health research, which in the past decade has been underpinned by a well-resourced infrastructure and dedicated organization (INVOLVE) (http://www.invo.org.uk/) to support PPI within the NIHR.

The NIHR commissioned a review of the state of PPI in research,^15^ which informed the INVOLVE decision to focus on three priorities. The first priority is to encourage more diversity, equity and inclusion. The second is to develop support, capacity building and learning and development for both public contributors and the research community. The third is community, network and partnership building. These priorities can be informed by the RAPPORT findings which also shed light on key areas of debate for policy makers to consider. These include maintaining a balance between the moral and methodological imperatives in evaluating PPI; seeking the most appropriate public contributors and how best to harness PPI skills and relationships developed over time; ensuring adequate resources and scale in PPI to support research whilst avoiding impersonally ‘industrializing’ PPI activities and finally, enabling optimal PPI contributions at all stages of research without adversely affecting research outcomes. Tensions are to be expected in PPI, but working through them in partnership can fuel novel research synergies.
